# Oculomotor, Vestibular, and Reaction Time Tests in Mild Traumatic Brain Injury

**DOI:** 10.1371/journal.pone.0162168

**Published:** 2016-09-21

**Authors:** Carey Balaban, Michael E. Hoffer, Mikhaylo Szczupak, Hillary Snapp, James Crawford, Sara Murphy, Kathryn Marshall, Constanza Pelusso, Sean Knowles, Alex Kiderman

**Affiliations:** 1 University of Pittsburgh, Department of Otolaryngology, Pittsburgh, PA, United States of America; 2 University of Miami, Miller School of Medicine, Department of Otolaryngology, Miami, FL, United States of America; 3 University of Miami, Miller School of Medicine, Department of Neurological Surgery, Miami, FL, United States of America; 4 University of Miami Sports Performance and Wellness Institute, Miami, FL, United States of America; 5 Madigan Army Medical Center, Department of Otolaryngology, Tacoma, WA, United States of America; 6 Naval Medical Center, San Diego, San Diego, CA, United States of America; 7 Neuro–Kinetics, Inc. Pittsburgh, PA, United States of America; Cleveland Clinic, UNITED STATES

## Abstract

**Objective:**

Mild traumatic brain injury is a major public health issue and is a particular concern in sports. One of the most difficult issues with respect to mild traumatic brain injury involves the diagnosis of the disorder. Typically, diagnosis is made by a constellation of physical exam findings. However, in order to best manage mild traumatic brain injury, it is critically important to develop objective tests that substantiate the diagnosis. With objective tests the disorder can be better characterized, more accurately diagnosed, and studied more effectively. In addition, prevention and treatments can be applied where necessary.

**Methods:**

Two cohorts each of fifty subjects with mild traumatic brain injury and one hundred controls were evaluated with a battery of oculomotor, vestibular and reaction time related tests applied to a population of individuals with mild traumatic brain injury as compared to controls.

**Results:**

We demonstrated pattern differences between the two groups and showed how three of these tests yield an 89% sensitivity and 95% specificity for confirming a current diagnosis of mild traumatic brain injury.

**Interpretation:**

These results help better characterize the oculomotor, vestibular, and reaction time differences between those the mild traumatic brain injury and non-affected individuals. This characterization will allow for the development of more effective point of care neurologic diagnostic techniques and allow for more targeted treatment which may allow for quicker return to normal activity.

## Introduction

Mild traumatic brain injury (mTBI) is an increasingly common public health concern that has garnered increased attention in both the lay press and medical literature. Neurosensory effects are among the most common sequela seen after mTBI, with balance-related findings chief among these. [1–8] The diagnosis of mTBI has always presented challenges. This is true in part because many of the symptoms are self-reported with variable intensity over time. Many of the most commonly used tests can only be performed in highly specialized labs with experienced providers. [9, 10] And even in these labs, there is a great deal of debate as to what constitutes definitive tests of balance dysfunction after mTBI. Moreover, the variety of tests utilized provides little help to standardized testing batteries now used as point of injury tests. [11] While such testing is important [12], in most cases the balance components are too easy to master even when an injury does exist to provide useful information. [13–15] In many cases, investigators have had to modify the vestibular components of the tests to achieve useful diagnostic information, but such work has not been standardized or well defined. [14, 15] It is quite possible that the current lab and field testing paradigms have not included the most sensitive and specific balance tests. [16]

One of the most interesting and promising areas includes examining oculomotor, vestibular and reaction time (OVRT) reflexes in response to a variety of visual and vestibular challenges. In this paper, we describe the use of a panel of OVRT tests performed with a set of infrared goggles on a rotational chair platform in the diagnosis of mTBI. This work has allowed us to identify some OVRT characteristics of mTBI and to show that a small subset of this panel can be utilized to achieve high specificity and sensitivity for the current diagnosis of mTBI. This objective OVRT pattern can then be utilized to help confirm diagnosis made by skilled medical professionals.

## Methods

This study and its written informed consent material were approved independently by the following IRB's: IRB at Naval Medical Center San Diego (NMCSD), IRB at Madigan Army Medical Center (MAMC), IRB at the University of Miami, Miller School of Medicine (UMMSM).

### Subject Selection

Individuals between the ages of 18 and 45 were recruited from the emergency rooms of one civilian and two military hospitals. Individuals were eligible for inclusion in the study if they had a diagnosis of mTBI from the emergency room. In particular, these individuals had a head injury and sequelae from the head injury with a Glasgow Comma Scale of 14 or greater and no loss of consciousness greater than 30 minutes. All individuals had a diagnosis of mTBI/concussion by an Emergency Room Staff Physician This group represents the spectrum of individuals seen in an emergency room with head injury. These individuals reported to the study sites at a scheduled time within six days of injury. The majority of subjects (52.8%) reported within 48 hours of injury; with 83.0% reporting within 96 hours. At the study site, individuals were assessed for mTBI and the presence of any exclusion criteria. Those who were not excluded were offered participation in the study. All those who agreed signed written informed consent that was approved by the IRB of each institution and kept in a study binder. Control subjects were recruited from volunteers at the locations where the study was being conducted. These individuals were also between the ages of 18–45 and were screened to assure that they had no active medical condition and did not have any history of significant mTBI, ear or balance disorders.

### Study Visits

All mTBI subjects underwent an initial visit, targeted to be within three days of injury, and were then requested to complete two follow up visits at one- and two-weeks post injury. Control subjects were requested to complete only the initial visit. At each visit, mTBI patients underwent a symptom profile (investigator-administered questionnaire), a functional gait index (FGI), a Dizziness Handicap Inventory (DHI), and the battery of OVRT tests detailed below. The control subjects completed the same assessments, with the exception of the FGI.

The battery of OVRT tests was conducted on a standard clinical I-Portal^®^ Neuro Otologic Test Center (I-Portal–NOTC, Neuro Kinetics Incorporated, 126 Gamma Drive, Pittsburgh, PA 15238) system located within a lightproof enclosure. Participants were secured into a computer-controlled rotational chair within 36 inches from a black featureless enclosure wall. Movements of each eye were recorded with integrated video cameras (100 frames per second) in head-mounted goggles using off-axis infrared lighting (940mn) and the black pupil technique for identifying the pupil centroid. Specific tests include in the battery are shown in table 1. A pattern of moving random dots, covering at least 90% of the visual field, was projected on the enclosure wall for optokinetic stimuli and a two axis, high speed servo-controlled galvanometer controls projection of a 650 nm 3 mm laser dot for fixation, pursuit and saccade target stimuli.

**Table 1:** Tests performed.

Test	Variables
Optokinetic	Left and Right Gain and Asymmetry for nystagmus beats
Smooth Pursuit–Horizontal/Vertical	Percent of Saccadic Intrusions, Initiation Time
Saccade-Random–Horizontal/Vertical	Saccade Onset Latency, Accuracy, Peak Velocity
Predictive Saccade	Point in cycle at which subject anticipates/predicts the fixed timing interval and dot position as well as percent of correct predictions
Anti-saccade Horizontal	Number of Pro-saccadic errors, correct anti-saccades, Latency, and Velocity
Self-paced Saccade	Saccades per second
Gaze Horizontal	Vertical peak and average slow phase velocity
Visual Reaction Time	Mean and Standard Deviation (SD) of Latency
Auditory Reaction Time	Mean and SD of Latency
Saccade and Reaction Time	Saccade Onset Latency, Accuracy, and Latency and SD for motor responses
Computer Controlled Rotation Head Impulse Test (crHIT)	Left and Right Gain and Asymetey
Sinusoidal Harmonic Acceleration (SHA)	Gain, Phase, and Asymmetry—High Frequencies
Visual Enhancement	Gain, Phase, and Asymmetry—High Frequencies
Visual Suppression	Gain, Phase, and Asymmetry—High Frequencies

In order to respond to visual and auditory stimuli, participants were provided left and right buttons located in the chair handles. These buttons were used to register a subject’s reaction to either visual or auditory stimuli, the latter of which were presented using an 85 decibel piezo-electric buzzer.

Two cohorts of fifty mTBI patients and two cohorts of one hundred control adults volunteered to participate and were included in this study analysis. Cohort 1 was accrued through August 2014; cohort 2 was accrued through October 2015. The ages for the participants ranged from 18 to 45 years and did not differ between cohorts or between the mTBI subjects and the controls within each cohort, either in mean values or age group distributions. For the combined cohorts, the age group distribution was 45% 18- to 25- year olds, 39% 26- to 35- year olds, and 16% 36- to 45- year olds. Gender representation for this study did not differ significantly between cohorts. The combined cohorts (mTBI and controls) included 226 (75.1%) males and 74 (24.9%) females. Females accounted for 33% of the individuals with mTBI and 20.5% of the control subjects across cohorts. All of the individuals in both cohorts were either high school graduates with some college education or college graduates. Given the neurologic nature of these tests, this level of education is sufficient for understanding the instructions provided by the examiner and differences between high school and college graduates would not be expected to affect this type of testing. All individuals in both cohorts reported that they had not suffered a TBI in the twelve months prior to the current visit (excepting the current mTBI by the mTBI subjects) and no individual in either cohort (mTBI or control) had a history of ever being hospitalized for TBI. As such we believe this population of subjects is representative of the general population at large.

All testing was performed in the MAMC or NMCSD clinical vestibular labs. The mTBI subjects were tested three times at equivalent intervals over an average of 18 days. The results presented in this paper are for the first testing sessions, which took place between 4–166 hours of the incident (mean = 2.6 days, SD = 1.6 days). The distribution of time between traumatic event and test was 52.8% within 48 hours, 76.4% within 72 hours, and 83% within 96 hours. The subject and test time demographic data are shown in more detail in Table 2. A successive, two cohort design was chosen for this case versus control cohort study, with replicability as a criterion to control for unknown confounding factors in biomarker studies with only a single cohort. [17]

**Table 2:** Characteristics of the subject population.

	Control Group		mTBI Group	
	Cohort 1	Cohort 2	Cohort1	Cohort 2
Gender (Females: Males)	25:75	19:81	21:29	12:38
Sample size (N)	100	100	50	50
Age (years, mean ± SD)	29.7±6.2	26.3±6.0	26.7±6.4	26.0±7.0
Symptom Score (22 item SCAT, 22 minus number symptoms, mean ± SD)	20.2±2.7	20.6±2.4	8.5±6.3	8.3±6.0
Symptom Severity (22 item SCAT, mean ± SD, max 132)	2.9±5.1	2.4±5.4	44.5±26.8	43.2±30.5
Time post-TBI (hours, mean ± SD)			58.1±35.6	66.6±39.6
Glasgow Coma Scale (mean ± SD)			15.0±0.0	14.8±1.0
Functional Gait Index (maximum 30, mean ± SD)			24.7±4.6	25.7±5.8
Dizziness Handicap Inventory Total Score (mean ± SD)			33.5±24.1	28.5±20.0
Trail Making Test A (sec, mean ± SD)			29.1±11.5	31.1±12.1
Trail Making Test B (sec, mean ± SD)			55.4±18.5	56.9±28.9

### Data Analysis

The goal was to identify OVRT performance metrics that differentiate between mTBI and control groups, as well as to create a model to enable us to accurately evaluate assessing mTBI neurologic status in patients. Logistic linear regression analysis was utilized to produce coefficients that could categorize subjects belonging to control or mTBI groups from the set of 105 measures that characterized each subject’s test performance. Maximum likelihood estimation was used in a step-wise algorithm to identify variables that could sufficiently separate the mTBI and control subject groups. Specifically, conditional regression (*p* = .05 to entry, *p* = .10 to removal) was performed using SPSS^®^ 21.0 or 24.0 software on 105 variables characterizing test performance.

Separate statistical models were replicated on data from each cohort of 50 mTBI and 100 control subjects, as well as data from combined cohorts. The ability to discriminate between study populations was assessed from receiver-operator characteristic (ROC) curves. The area under the curve, sensitivity (true positive rate) and specificity (true negative rate) were calculated to assess the classification. Leave-one-out and in-out sample 70%/30% cross-validation showed that the models estimates for classification are stable.

## Results

In this study, we tested the hypothesis that videonystagmography and reaction time responses can be used to quantify the profile of OVRT deficits that form the basis for an objective neurologic diagnosis of mild traumatic brain injury. This documents a small set of non-invasive, reliable, and objective measurements from a battery of OVRT tests that provide metrics to identify objectively the individuals with mTBI.

**Fig 1:**
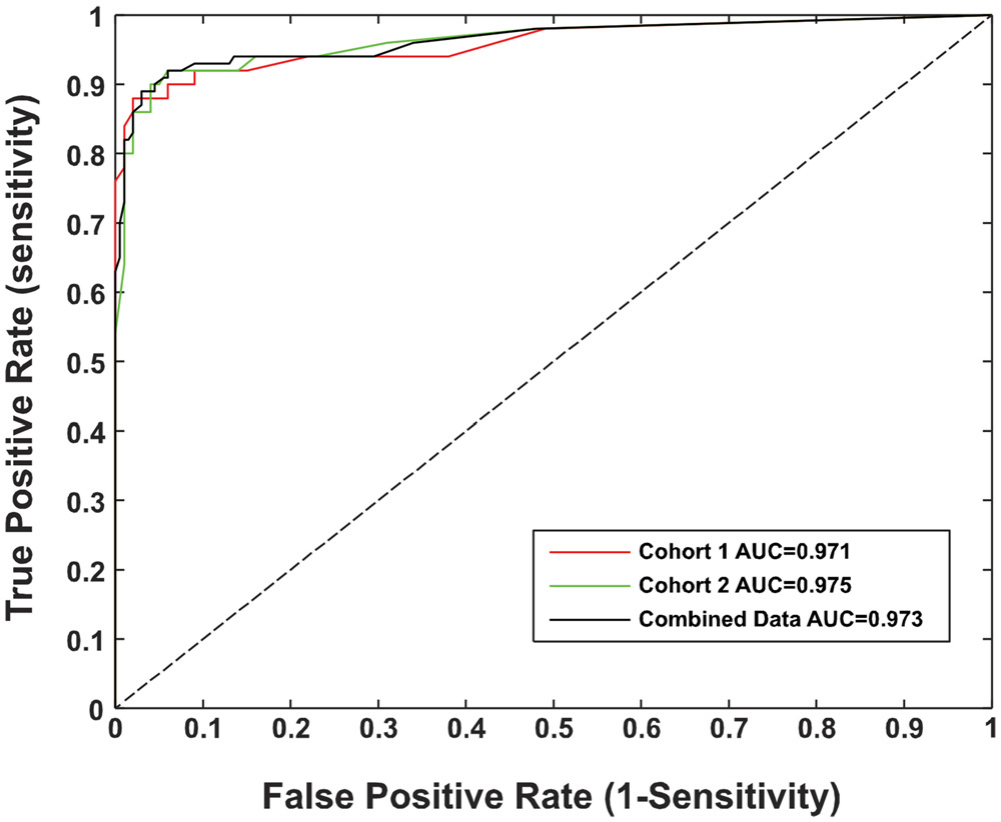
ROC curve for Individual Cohorts and Combined Group. ROC curves for the first cohort of subjects with mTBI (red), second cohort of subjects with mTBI (green), and the combination of both groups (black).

Characteristics of the subject population can be seen in Table 2. The two cohorts did not differ in functional test results; impairment was in the mild range relative to population norms for the FGI, DHI and trail making tests. The OVRT data from each cohort were subjected separately by step-wise logistic regression analysis. The parameter estimates for logistic regression models of each cohort and the combined data are summarized in Table 3 and the sensitivities and specificities are summarized in Table 4. These analyses identified four metrics from different tasks as significant indicators of a mTBI in both cohorts (p<0.001 for the model). The overall prosaccade error rate is a measure of the ability to inhibit erroneous saccades, while the predictive saccade performance measures the error rate of saccade response to a predictable stimuli. The absolute gain symmetry and average gain of eye movements on the computer controlled rotational head impulse test (crHIT) are measures of high frequency horizontal vestibulo-ocular reflex performance that can detect enter unidirectional or bidirectional deficits. The predictive saccade metric was the number of predictive saccades (beginning less than 50 ms after target onset) for presentation of alternating ± 10 degree targets at a fixed interval of 650 ms. The estimated parameters were virtually identical for the two cohorts, with a specificity of at least 97% (at the default 0.5 cutoff), meaning that they correctly rejected at least 97 of the 100 control subjects in each of the respective cohorts as not belonging to the mTBI group. The sensitivity was at least 88%, indicating that at least 44/50 mTBI patients were ‘correctly’ identified. For the combined cohorts, the specificity was 97.5% and the selectivity 89%. The ROC plots for all three models are shown in Fig 1. The areas under the curve (AUC) of the ROC characteristics exceeded 0.97 for the cohorts and the combined data. In-out sample (70%/30%) validation for the combined data revealed that the AUC for this validation model was 0.9765; model sensitivity was 90.9% and specificity 98.5%. Leave-one-out validation revealed a model sensitivity of 87% and specificity 97%. Hence, these four metrics provide robust and objective identification of the group with acute mTBI.

**Table 3:** Parameters for logistic regression models and significance levels. (* p<0.05, ** p<0.01, *** p<0.001).

	Cohort 1		Cohort 2		Combined	
Parameter (coefficient)	Estimate ± SE	Wald	Estimate ± SE	Wald	Estimate ± SE	Wald
Prosaccade error (%)	0.129±0.034	13.97***	0.107±0.028	14.91***	0.117±0.021	31.00***
crHIT absolute gain symmetry	0.824±0.231	12.79***	1.099±0.280	15.43***	0.9297±0.166	31.32***
crHIT average gain	-32.216±8.901	13.10***	-36.603±10.039	13.29***	-32.058±6.025	28.30***
Predictive Saccades (number)	-0.190±0.077	6.18*	-0.204±0.071	8.26**	-0.195±0.050	15.51***
Intercept	26.212±8.024	10.67**	30.895±8.956	11.90***	26.456±5.461	23.47***

**Table 4:** Sensitivities and specificities.

	True Positive (Sensitivity)	True Negative (Specificity)	Correct	ROC AUC
**Cohort 1: Data**	88%	99%	95.3%	0.9714
**Cohort 2: Data**	92%	98%	96.0%	0.9752
**Combined: Data**	89.0%	97.5%	94.7%	0.9727
70/30 in-out sample	90.9%	98.5%	97%	0.9765
Leave one out	87%	97%	93.7%	

Summary statistics for these individual metrics (Table 5) were highly reproducible across cohorts. The overall prosaccade error rate (a measure of impaired inhibition of saccades) was elevated in subjects with mTBI (p<0.001 for either cohort and for the combined data). The crHIT gain was more asymmetric in the subjects with mTBI (p<0.001 for either cohort and for the combined data). The crHIT average gain (average of leftward and rightward responses) was also reduced significantly in subjects with acute mTBI (p<0.001 for either cohort and for the combined data). Significant reductions were also present in the mTBI groups for predictive saccade performance (p<0.001 for either cohort and for the combined data) and the saccadic reaction time latency (p<0.01 for either cohort and for the combined data).

**Table 5:** Summary statistics for each cohort and combined group.

	Cohort 1		Cohort 2		Combined	
Variables	Control	mTBI	Control	mTBI	Control	mTBI
Prosaccade error (% responses)	12.8±12.7	**31.2±20.4**	12.8±10.5	**37.3±26.4**	12.8±11.6	**34.2±23.7**
crHIT absolute gain symmetry	1.8±1.2	**5.5±4.4**	1.6±1.3	**6.2±5.0**	1.7±1.2	**5.9±4.7**
crHIT average gain	0.96±0.04	**0.86±0.12**	0.97±0.04	**0.82±0.12**	0.96±0.04	**0.84±0.12**
Predictive Saccades (number)	14.5±4.8	**9.6±5.8**	15.4±4.1	**11.0±6.0**	14.9±4.4	**10.3±5.9**

**Fig 2:**
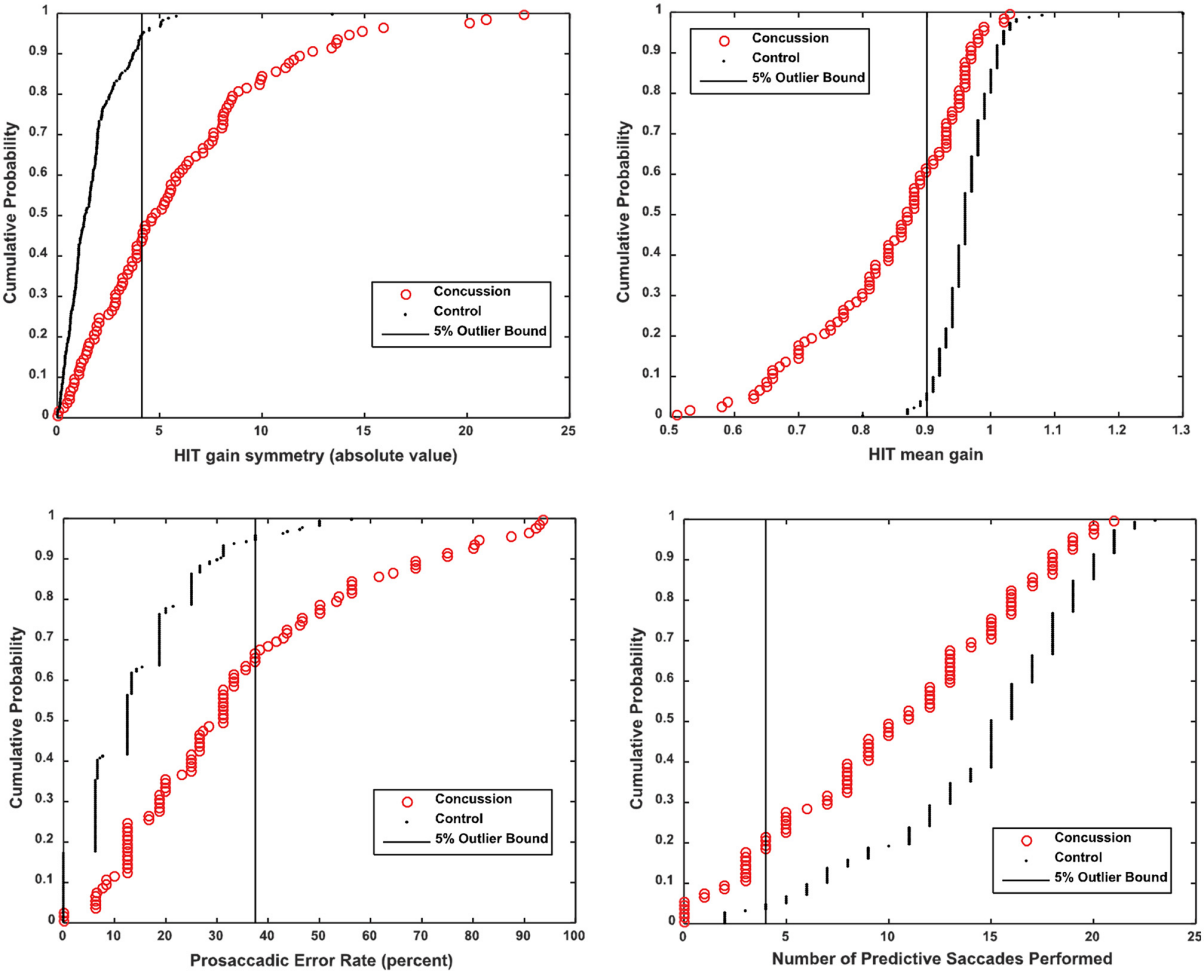
Cumulative distribution functions are shown for the four metrics in the logistic regression model, 89% sensitivity and 97.5% specificity. Concussion (red) plotted with controls (black). The vertical line in each graph demarcates the location of the upper or lower 5% cutoff for control subjects.

To this point, we have reported that a weighted linear combination of these metrics differentiated mTBI subjects from controls. It is also instructive to examine the patterns displayed by individual mTBI subjects. Ninety-two of the subjects were outside the 95% range of the control population on one or more of the four metrics in the logistic regression model. The cumulative distribution functions for the combined cohorts are shown in Fig 2. The most prevalent occurrence outside the 95% control range was the crHIT average gain (62/100 mTBI subjects, less than 0.9), followed by the absolute gain symmetry (56/100 mTBI subjects, greater than 4.14%), the error rate for the anti-saccade saccade task (36/100 mTBI subjects; at least 37.5% error), 22/100 for the increased saccade onset latency (at least 0.26 sec) and 22/100 for the number of predictive saccades. Twenty-six of these subjects had only one metric outside of the 95% control range (9 for a high crHIT gain asymmetry, 7 for a low crHIT average gain, 6 for an elevated error rate the in the anti-saccade task, 1 for a slow saccadic latency and 3 for a low level of predictive saccade performance). The remaining 66 subjects showed two or more metrics outside the 95% range for controls. The combinations are listed in Table 6. Twenty-nine mTBI subjects had three or more or more metrics outside the 95% range for controls. For example, 17/19 subjects with outlier scores for the prosaccadic error rate and crHIT gain symmetry also had an outlier finding for the crHIT average gain.

**Table 6:** Prevalence of paired combinations of metrics outside the 95% control performance levels in subject with acute mTBI.

	crHIT gain symmetry	crHIT average gain	Predictive Saccades
Prosaccade error	19	25	9
crHIT gain symmetry		39	12
crHIT average gain			12

It also important to note that the performance of mTBI subjects did not differ from controls on test metrics such as gain and phase of the low frequency horizontal vestibulo-ocular reflex, horizontal saccade accuracy, and the phase of the velocity of horizontal smooth pursuit of visual target. These normal results argue against a lack of attention or motivation as a confounding factor in this population.

## Discussion

This study identifies metrics from three OVRT tests that identify patients who have sustained a recent mild mTBI; furthermore, we are able to distinguish them from uninjured control subjects with greater than 97% sensitivity and 89% specificity. It is important to note that these measures discriminate mTBI patients from controls: (1) without a requirement for baseline test data before the concussive event, (2) independent of gender, and (3) for testing within a window of at least 4 days after the event. Hence, they can form the basis for a robust, objective screening test for acute mTBI.

Two main caveats regarding diagnosis and study design qualify the reported sensitivity and specificity findings from these test populations. Firstly, the diagnosis of TBI, particularly mTBI, is far from straightforward. [11, 12] In this study population, mTBI was defined by (1) exposure to a documented concussive event, (2) either an alteration or momentary loss of consciousness, and (3) the presence of a defined set of post-concussive symptoms. Because there is not a distinctive, objective threshold for diagnosing mTBI, it is possible that the diagnosis may include a few individuals who will not display objective signs on further examination. Secondly, there was no baseline testing of the control population and the only medical history was a self-report. Hence, it is possible that the control population may include individuals with chronic, asymptomatic sequelae of mTBI. Given these factors, we consider a sensitivity of 0.975 and selectivity of 0.89 to be strong results for a test battery of only four metrics that does not require pre-injury baseline data.

### Individual Test Significance

Because the different metrics that discriminate the acute mTBI group from the control subjects are statistically uncorrelated, they can collectively give insight into combinations of underlying dysfunction in those presenting with acute mTBI. It is instructive to begin with a consideration of their potential significance individually. The anti-saccade task is one assessment of the core executive function of response inhibition. [18] The increased prosaccadic error rate during the anti-saccade task likely reflects impaired inhibitory contributions of the influences of several frontal cortical regions (frontal eye fields, supplemental eye fields and dorsolateral prefrontal cortex) and the basal ganglia on GABAergic output from substantia nigra, pars reticulata to the superior colliculus and thalamus. [18] This inhibitory contribution appears to be critical for suppressing the prosaccade. Like other saccades, an antisaccade is thought to be programmed in the frontal cortex. The difference in antisaccade error rate between the control and mTBI groups is a further indication that the higher level performance of these pathways is sensitive to acute mTBI.

The head impulse test gain and gain symmetry provide measures of high frequency vestibulo-ocular reflex function. It is presumed to be a measure of functional performance of a network that includes the vestibular periphery, the vestibular nuclei and related cerebellar connections, and direct projections from the vestibular nuclei to the abducens, trochlear and oculomotor nuclei. In addition to peripheral vestibular insults, abnormal HIT gains have been reported for conditions such as brain stem strokes and cerebellar ataxia.[19, 20]

The impaired ability to generate predictive (or anticipatory saccades) was observed. The ability of normal subjects to switch from a reactive to a predictive saccade generation mode has long been noted in the literature.[21, 22] Predictive saccade performance is impaired in neurologic disorders such as Parkinson’s disease, Parkinson’s disease dementia, and dementia with Lewy bodies.[21, 22] Saccadic performance factors that may contribute to the observed decrement in making predictive saccades will be the subject of a future communication.

### Differentiating mTBI from Controls

The set of four uncorrelated metrics appears to differentiate mTBI patients from controls on the basis of performance of neural networks that involve: (1) processing capacity of prefrontal cortical circuits and (2) posterior fossa processing associated with motion detection and ocular responses; however, we cannot disentangle how the findings indicate both direct and ‘downstream’ effects of concussive events, as well as evolving functional consequences of recovery and compensation on cognitive reserve.[21, 22] The anti-saccade task error rate and the predictive (or anticipatory) saccade performance need to be considered as proxies for cognitive reserve.

We have described a set of elements taken from a larger test battery of oculomotor and vestibular tests performed on a larger balance testing device located in specialized tertiary care facilities. The tests performed with this device each provide information about OVRT neurologic function. The tests are most conveniently considered as functional test batteries. Combining all the tests produces a slight increase in both values yielding 89% sensitivity and 95% specificity. Moreover, these last values can be achieved with a subset the combined tests (anti-saccade testing, predictive saccade testing, and crHIT). As such this work provides initial information to help solve the dilemma facing those who study mTBI. The combination can be used as one secure element in any test battery being performed in the clinic setting to diagnose mTBI. Also it should be noted that this particular subset of three tests can be performed without the large tertiary center device and without specialized medical training. In this way, an initial quantitative assessment of neurologic status can be performed near the point of injury to and this device can be used to help medical professionals make diagnosis in a variety of settings away from traditional clinics. This initial work could profit from further exploration. The use of these tests on subjects in an at risk population followed over time would help to further validate this model. Such work is already underway in our lab.
